# 'Splenic switch-off' to detect adenosine understress; a novel method to improve test sensitivity

**DOI:** 10.1186/1532-429X-16-S1-O1

**Published:** 2014-01-16

**Authors:** Charlotte Manisty, David P Ripley, Gaby Captur, Charles Peebles, Timothy C Wong, Erik B Schelbert, Anna S Herrey, John P Greenwood, James Moon

**Affiliations:** 1 Heart Hospital Imaging Centre and Imperial College, London, UK; 2 University Hospital, Southampton, Southampton, UK; 3 Heart Hospital Imaging Centre and University College, London, UK; 4 Multidisciplinary Cardiovascular Research Centre (MCRC) & Leeds Institute of Genetics, Health and Therapeutics, University of Leeds, Leeds, UK; 5 UPMC Cardiovascular Magnetic Resonance Center, Heart and Vascular Institute, University of Pittsburgh, Pittsburgh, Pennsylvania, USA; 6 Heart Hospital Imaging Centre and Royal Free Hospital, London, UK

## Background

The sensitivity of adenosine perfusion CMR is reduced by false negative scans, with up to 50% resulting from inadequate pharmacological stress. Without a robust physiological marker for adequate myocardial hyperaemia, this false negative rate is difficult to address. We observed that splenic perfusion is markedly attenuated with adenosine - compared both to rest and to myocardial perfusion. In this collaborative multi-center study, we investigate the pharmacology of 'splenic switch-off', and evaluate its potential clinical utility as a marker of inadequate stress in adenosine perfusion imaging.

## Methods

We assessed splenic perfusion in 4 cohorts acquired in 4 separate CMR units using 3 different pharmacological stressors. This study included: • Verification cohort of 50 adenosine perfusion scans (London, UK); to determine if splenic perfusion is consistently switched-off with adenosine. • 2 comparison cohorts using alternative pharmacological stressors (25 dobutamine scans; Southampton, UK and 25 regadenoson scans; Pittsburgh, USA); to assess whether generic stress (or only adenosine) causes splenic switch-off. • Clinical utility cohort of 100 adenosine scans (35 false and 65 true negative) from the CE-MARC trial (Leeds, UK); to assess whether failure of splenic switch-off could be a useful clinical indicator of inadequate stress.

## Results

**Figure 1:**
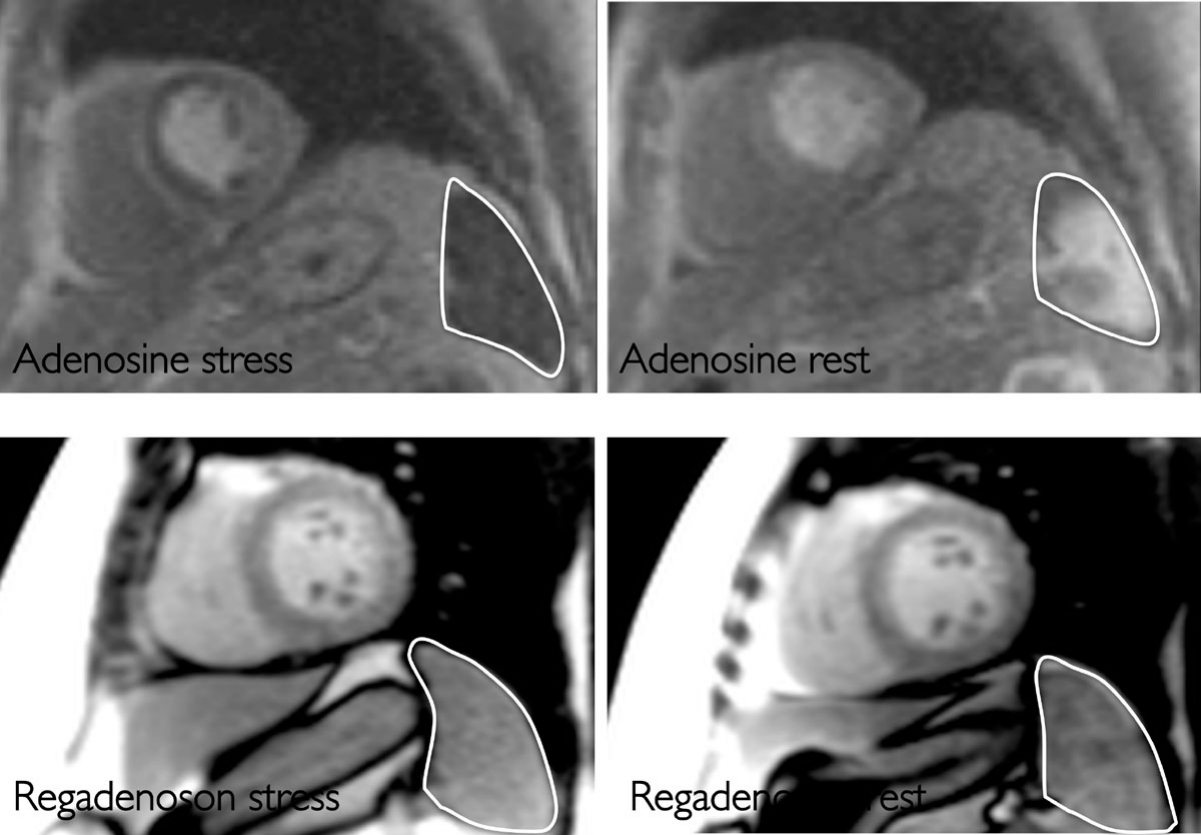
**Splenic perfusion at stress and rest with adenosine (upper panels) and regadenoson (lower panels), showing splenic switch-off with adenosine only**.

**Figure 2:**
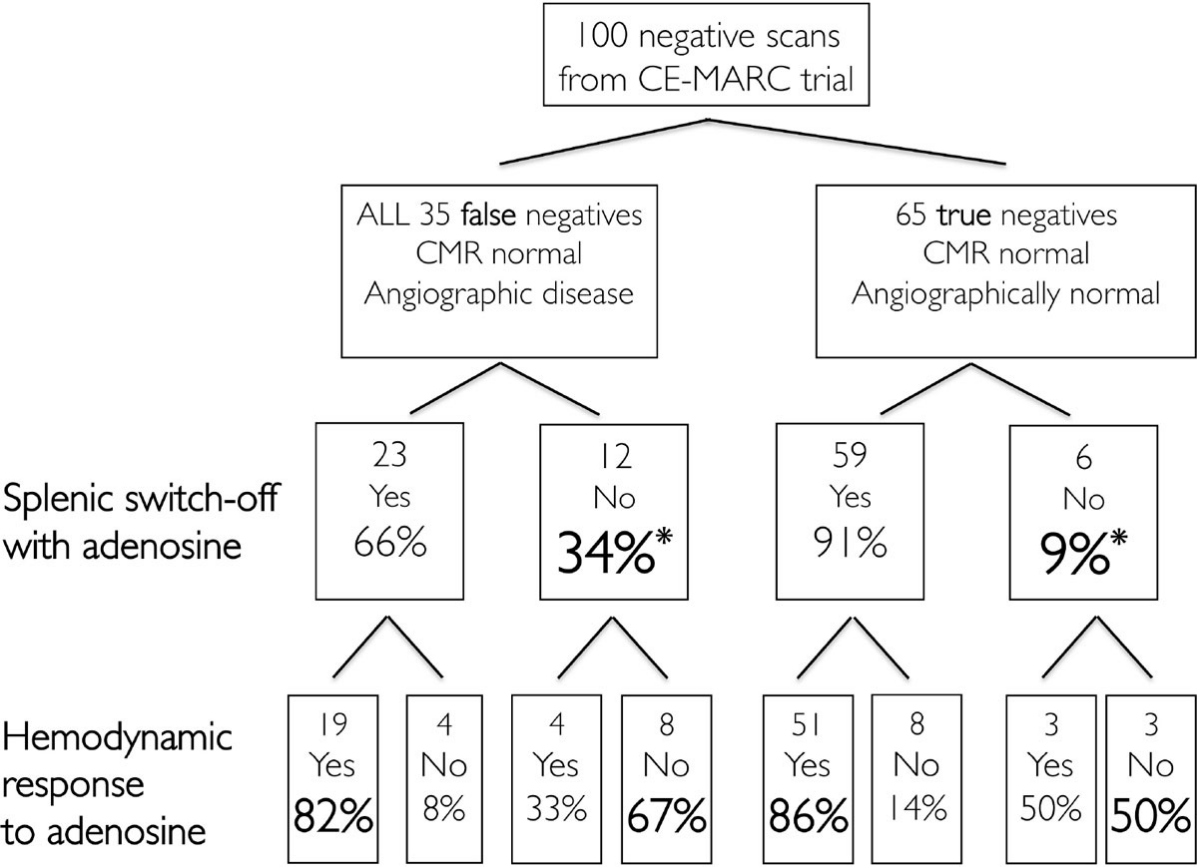
**Data from CE-MARC trial to assess splenic and hemodynamic responses to adenosine There were significantly more patients with false negative CMR perfusion scans who failed to switch-off splenic perfusion with adenosine (indicating inadequate pharmacological stress) in comparison to those with true negative scans**. Concordance was good between hemodynamic and splenic responses to adenosine. *p = 0.0027.

The spleen was visible in 98.5% of scans and grading of splenic perfusion was concordant between 2 blinded observers, κ = 0.84. Splenic switch-off occurred in 92% of adenosine studies acquired in London, but did not occur either with dobutamine or regadenoson perfusion studies, Figure 1. Measuring perfusion semi-quantitatively using signal intensity, splenic perfusion with adenosine stress was significantly lower than at rest (8.1 ± 9 versus 33.3 ± 19 arbitrary units, p < 0.0001), in contrast to with regadenoson where it increased significantly (123.7 ± 56.7 versus 144.6 ± 59.2 au, p = 0.003). With dobutamine (where only stress images were acquired), splenic perfusion was greater than myocardial (54.1 ± 1 versus 67.6 ± 25.2 au, p = 0.0005), again in contrast to adenosine. Within the CE-MARC cohort, patients with false negative CMR scans had a 36% rate of failed splenic switch-off. By contrast, the true negative group had a 9% rate (p = 0.0027 for difference), Figure 2. Splenic response to adenosine was concordant with haemodynamic response in 81% of subjects.

## Conclusions

Splenic switch-off with adenosine is a new observation, and although a drug-specific effect, can be assessed in nearly all scans. Rescanning individuals with failure of splenic switch-off would reduce false negative scans by a third, but it may be that up to 1 in 11 of all adenosine perfusion patients are understressed. Further work is needed on this important sign.

## Funding

CM is an NIHR Clinical Lecturer.

